# Decorated and Encapsulated: Virus-Like Particles Against Viral Infections

**DOI:** 10.3390/vaccines9030273

**Published:** 2021-03-18

**Authors:** Vladimir Temchura

**Affiliations:** Institute of Clinical and Molecular Virology, University Hospital Erlangen, Friedrich-Alexander-University Erlangen-Nürnberg, 91054 Erlangen, Germany; vladimir.temchura@fau.de

Despite great progress in the field of vaccine development, outbreaks of emerging pathogens and insufficient immunogenicity of some licensed vaccines call for the development of novel technologies in rational vaccine design. The intrinsic properties of virus-like particles (VLP) together with modern genetic and biochemical tools turn nanoparticle-based vaccines into a flexible platform for the on demand modulation of both the strength and quality of antiviral immune responses. This editorial, aims to summarize the latest scientific and technical developments in the field of antiviral VLP vaccines. Such advancements have been collected in the Special Issue entitled “Virus-Like Particle and Nanoparticle Vaccines against Viral Infections”.

VLPs are the ideal nano-vaccine system, as they harness the power of the evolved viral structure—which is naturally optimized for interaction with the immune system—but avoid the infectious components [1]. Further biotechnological optimization of VLPs is exceptionally innovative and includes multiple approaches [2]. The review by Keikha et al. provides a compact overview of the nanobiotechnology involved in immunology and vaccination. The authors outline the major types of nano-based vaccines with a particular focus on VLP nano-vaccines [3].

Application of antiviral VLP vaccine candidates also creates new challenges. Zhang et al. not only summarized recent advances in the development of VLP-based flavivirus vaccines, but also critically discussed a number of points that still need to be addressed. In their multifaceted review, the authors also illustrate potential strategies to improve the efficacy of VLP-based flavivirus vaccines and even propose to apply flavivirus VLPs as tools for viral detection and antiviral drug screening [4].

The SARS-CoV-2 outbreak and the lessons learnt from other respiratory viruses have stirred up discussions concerning the role of generated memory T-cell pools in the protective antiviral immune responses [5,6,7]. In this Special Issue, Nicolas W. Lukas and Carrie-Anne Malinczak discussed immune responses to respiratory viruses and how the cellular immune response may be harnessed in order to produce more promising vaccine candidates for viruses that have consistently been difficult to target. This detailed review refers to the history of vaccine-enhanced disease, surveys the immune responses to respiratory viruses and highlights exciting aspects of novel safety vaccination approaches against respiratory viral infections [8].

Even with numerous modern technological and scientific advances, the challenge of vaccinating against human influenza A virus (IAV) lies in the constantly changing nature of the virus itself (antigenic drift and shift). This has forced frequent updates of current IAV vaccines, which need to be administered annually [9]. A universal flu vaccine (UFV) that provides long-lasting protection against both seasonal and emerging pandemic IAV strains is, thus, urgently needed. For such UFV candidates, a stable trimeric influenza hemagglutinin stem (HA_stem_) might be a promising broadly protective immunogen [10]. In their research article, Susan Thrane and colleagues described the development of a UFV candidate consisting of the HA_stem_ trimer displayed on the surface of rigid capsid-like particles (CLP). Upon mixing of the antigen and CLP, stable antigen:CLP complexes are formed between the reactive tag and catcher protein, which are genetically fused to the antigen and CLP subunit protein, respectively. A single dose of the CLP-HA_stem_ vaccine provided protection against both homologous and heterologous influenza virus challenge in mice and demonstrated that this vaccine candidate outperformed another vaccine based on the soluble HA_stem_ trimer [11]. Although further studies will be needed to fully reveal the mechanism underlying this enhanced efficacy, these results support the use of the presented Tag/Catcher platform in the development of vaccines targeting other trimeric viral antigens.

Envelope glycoprotein (Env) trimers of the human immunodeficiency virus type 1 (HIV-1) represent another prominent type of viral target antigen, as designing a vaccine against HIV-1 still remains one of the greatest challenges for modern vaccinology. Since antibody binding to native-like Env trimers can result in HIV-1 neutralization, VLP technology offers a platform to test the hypothesis that native Env trimers presented in membranes may be useful for inducing neutralizing antibodies in a vaccine setting. So far, unmodified “wild type” HIV-1 VLPs have not fulfilled this potential [12]. Christopher A. Gonelli and co-authors increased the incorporation of HIV-1 Env into mature VLPs (mVLP) by replacing the Env transmembrane and cytoplasmic tail domains with those of influenza hemagglutinin. Furthermore, Env was stabilized on the VLP surface by introducing SOSIP mutations which are typically employed to stabilize soluble Env trimers. The resulting mVLP efficiently presented neutralizing antibody epitopes, while minimizing exposure of non-neutralizing antibody binding sites [13]. Therefore, mVLP displaying stabilized Env trimers are promising immunogens and warrant further investigation, especially when expressed in vivo from nucleic acid vaccine vectors.

A flurry of research activity during the most recent Zika virus (ZIKV) epidemic (2015) stimulated the development of a number of vaccine candidate prototypes [14]. As pregnant women and women of child-bearing age are the potential key target group for vaccination, ZIKV vaccine safety is an issue of major concern. Using a recently described chimeric flavivirus vaccine technology based on the novel insect-specific Binjari virus (BinJV) [15], Hazelwood et al. generated a ZIKV vaccine (BinJ/ZIKA-prME) and illustrated its ability to protect mice against fetal brain infection without the use of adjuvants [16]. BinJ/ZIKV-prME virus particles are structurally and antigenically authentic to mature ZIKV, but do not replicate in vertebrate cells [15] and might be formally attributed as a multiplication-defective VLP vaccine. BinJ/ZIKA-prME, therefore, presents attractive characteristics of a vaccine intended for use in pregnant women or women of child-bearing age. Another potential safety concern for ZIKV vaccines, antibody-dependent enhancement of dengue virus infections, was also not evident after BinJ/ZIKA-prME vaccination of mice [16].

Recently, various applications of nanotechnology have started to find their way in to the veterinary sector and now increasingly invade the production of veterinary vaccines [17]. Foot-and-mouth disease virus (FMDV) can cause highly contagious foot-and-mouth disease (FMD) of cloven-hoofed animals [18]. An experimental FMDV VLP (VLP_FMDV_) vaccine induced a sustained humoral immune response, but was not sufficient to provide 100% protective immunity in the setting of a large animal infection model [19]. In order to improve immunogenicity of VLP_FMDV_, Woo Sik Kim et al. manufactured a new vaccine by encapsulation of VLP_FMDV_ in monophosphoryl lipid A and liposomes (MPL/DDA-VLP_FMDV_). The MPL/DDA-VLP_FMDV_ formulation demonstrated the ability to (i) induce strong antigen-specific Th1 and Th17 immune responses, (ii) generate antigen-specific multifunctional CD4^+^ and CD8^+^ memory T cells, and (iii) increase antigen-specific antibody titers [20]. Considering the otherwise low immunogenicity of VLP_FMDV_, MPL/DDA encapsulation might be an excellent adjuvant not only for veterinary VLP_FMDV_ vaccines, but also for human antiviral VLP-based vaccine candidates.

Together with the success of the licensed VLP-based vaccines, the latest scientific and technical achievements collected in this Special Issue offer encouragement towards achieving the production of effective, safe and affordable antiviral VLP-based vaccines.

## Data Availability

Not applicable.

## References

[1] Zhao L., Seth A., Wibowo N., Zhao C.-X., Mitter N., Yu C., Middelberg A.P. (2014). Nanoparticle vaccines. Vaccine.

[2] Frietze K.M., Peabody D.S., Chackerian B. (2016). Engineering virus-like particles as vaccine platforms. Curr. Opin. Virol..

[3] Keikha R., Daliri K., Jebali A. (2021). The Use of Nanobiotechnology in Immunology and Vaccination. Vaccines.

[4] Zhang N., Li C., Jiang S., Du L. (2020). Recent Advances in the Development of Virus-Like Particle-Based Flavivirus Vaccines. Vaccines.

[5] Ledford H. (2020). What the immune response to the coronavirus says about the prospects for a vaccine. Nat. Cell Biol..

[6] Shah V.K., Firmal P., Alam A., Ganguly D., Chattopadhyay S. (2020). Overview of Immune Response During SARS-CoV-2 Infection: Lessons From the Past. Front. Immunol..

[7] Poland G.A., Ovsyannikova I.G., Kennedy R.B. (2020). SARS-CoV-2 immunity: Review and applications to phase 3 vaccine candidates. Lancet.

[8] Lukacs N.W., Malinczak C.-A. (2020). Harnessing Cellular Immunity for Vaccination against Respiratory Viruses. Vaccines.

[9] Kim H., Webster R.G., Webby R.J. (2018). Influenza Virus: Dealing with a Drifting and Shifting Pathogen. Viral Immunol..

[10] Impagliazzo A., Milder F., Kuipers H., Wagner M.V., Zhu X., Hoffman R.M., Van Meersbergen R., Huizingh J., Wanningen P., Verspuij J. (2015). A stable trimeric influenza hemagglutinin stem as a broadly protective immunogen. Science.

[11] Thrane S., Aves K.-L., Uddbäck I.E.M., Janitzek C.M., Han J., Yang Y.R., Ward A.B., Theander T.G., Nielsen M.A., Salanti A. (2020). A Vaccine Displaying a Trimeric Influenza-A HA Stem Protein on Capsid-Like Particles Elicits Potent and Long-Lasting Protection in Mice. Vaccines.

[12] Tong T., Crooks E.T., Osawa K., Robinson J.E., Barnes M., Apetrei C., Binley J.M. (2014). Multi-parameter exploration of HIV-1 virus-like particles as neutralizing antibody immunogens in guinea pigs, rabbits and macaques. Virology.

[13] Gonelli C., King H., Mackenzie C., Sonza S., Center R., Purcell D. (2021). Immunogenicity of HIV-1-Based Virus-Like Particles with Increased Incorporation and Stability of Membrane-Bound Env. Vaccines.

[14] Thomas S.J., Barrett A. (2020). Zika vaccine pre-clinical and clinical data review with perspectives on the future development. Hum. Vaccines Immunother..

[15] Hobson-Peters J., Harrison J.J., Watterson D., Hazlewood J.E., Vet L.J., Newton N.D., Warrilow D., Colmant A.M.G., Taylor C., Huang B. (2019). A recombinant platform for flavivirus vaccines and diagnostics using chimeras of a new insect-specific virus. Sci. Transl. Med..

[16] Hazlewood J.E., Rawle D.J., Tang B., Yan K., Vet L.J., Nakayama E., Hobson-Peters J., Hall R.A., Suhrbier A. (2020). A Zika Vaccine Generated Using the Chimeric Insect-Specific Binjari Virus Platform Protects against Fetal Brain Infection in Pregnant Mice. Vaccines.

[17] El-Sayed A., Kamel M. (2018). Advanced applications of nanotechnology in veterinary medicine. Environ. Sci. Pollut. Res..

[18] Grubman M.J., Baxt B. (2004). Foot-and-Mouth Disease. Clin. Microbiol. Rev..

[19] Xiao Y., Chen H.-Y., Wang Y., Yin B., Lv C., Mo X., Yan H., Xuan Y., Huang Y., Pang W. (2016). Large-scale production of foot-and-mouth disease virus (serotype Asia1) VLP vaccine in Escherichia coli and protection potency evaluation in cattle. BMC Biotechnol..

[20] Kim W.S., Zhi Y., Guo H., Byun E.-B., Lim J.H., Seo H.S. (2020). Promotion of Cellular and Humoral Immunity against Foot-and-Mouth Disease Virus by Immunization with Virus-Like Particles Encapsulated in Monophosphoryl Lipid A and Liposomes. Vaccines.

